# Influence of Extractive Solvents on Lipid and Fatty Acids Content of Edible Freshwater Algal and Seaweed Products, the Green Microalga *Chlorella kessleri* and the Cyanobacterium *Spirulina platensis*

**DOI:** 10.3390/molecules19022344

**Published:** 2014-02-21

**Authors:** Jarmila Vavra Ambrozova, Ladislava Misurcova, Robert Vicha, Ludmila Machu, Dusan Samek, Mojmir Baron, Jiri Mlcek, Jiri Sochor, Tunde Jurikova

**Affiliations:** 1 Department of Food Analysis and Chemistry, Faculty of Technology, Tomas Bata University in Zlin, nam. T. G. Masaryka 5555, Zlin CZ-760 01, Czech Republic; E-Mails: ambrozova@ft.utb.cz (J.V.A.); lmachu@ft.utb.cz (L.M.); dsamek@ft.utb.cz (D.S.); mlcek@ft.utb.cz (J.M.); 2 Department of Chemistry, Faculty of Technology, Tomas Bata University in Zlin, nam. T. G. Masaryka 5555, Zlin CZ-760 01, Czech Republic; E-Mail: rvicha@ft.utb.cz; 3 Department of Viticulture and Enology, Faculty of Horticulture, Mendel University in Brno, Valticka 337, Lednice CZ-691 44, Czech Republic; E-Mails: MojmirBaron@seznam.cz (M.B.); sochor.jirik@seznam.cz (J.S.); 4 Department of Natural and Informatics Sciences, Faculty of Central European Studies, Constantine the Philosopher University in Nitra, Drazovska 4, Nitra SK-949 74, Slovak Republic; E-Mail: tjurikova@ukf.sk

**Keywords:** algae, seaweed, lipid, fatty acid

## Abstract

Total lipid contents of green (*Chlorella pyrenoidosa*, C), red (*Porphyra tenera*, N; *Palmaria palmata*, D), and brown (*Laminaria japonica*, K; *Eisenia bicyclis*, A; *Undaria pinnatifida*, W, WI; *Hizikia fusiformis*, H) commercial edible algal and cyanobacterial (*Spirulina platensis*, S) products, and autotrophically cultivated samples of the green microalga *Chlorella kessleri* (CK) and the cyanobacterium *Spirulina platensis* (SP) were determined using a solvent mixture of methanol/chloroform/water (1:2:1, v/v/v, solvent I) and *n*-hexane (solvent II). Total lipid contents ranged from 0.64% (II) to 18.02% (I) by dry weight and the highest total lipid content was observed in the autotrophically cultivated cyanobacterium *Spirulina platensis*. Solvent mixture I was found to be more effective than solvent II. Fatty acids were determined by gas chromatography of their methyl esters (% of total FAMEs). Generally, the predominant fatty acids (all results for extractions with solvent mixture I) were saturated palmitic acid (C16:0; 24.64%–65.49%), monounsaturated oleic acid (C18:1(n-9); 2.79%–26.45%), polyunsaturated linoleic acid (C18:2(n-6); 0.71%–36.38%), α-linolenic acid (C18:3(n-3); 0.00%–21.29%), γ-linolenic acid (C18:3(n-6); 1.94%–17.36%), and arachidonic acid (C20:4(n-6); 0.00%–15.37%). The highest content of ω-3 fatty acids (21.29%) was determined in *Chlorella pyrenoidosa* using solvent I, while conversely, the highest content of ω-6 fatty acids (41.42%) was observed in *Chlorella kessleri* using the same solvent.

## 1. Introduction

Many recent studies have focused on the chemical composition of seaweeds, their positive contributions to human health and their possible usage as foodstuffs. Seaweed consumption has a long tradition in Asian countries and has increased in European countries in recent years, therefore approximately 20 species of edible algae are now available on the European market. Nowadays, freshwater algae and seaweeds have been extensively studied as good sources of many bioactive substances such as fatty acids, sterols, proteins, amino acids, minerals, polysaccharides or selected halogenated compounds, with extensive health benefit activities [1,2,3,4,5,6]. Freshwater algae and seaweeds, like fruits and vegetables, exhibit antibacterial, anti-inflammatory, anticancer, antiviral, anticoagulant, and other interesting properties [7,8,9]. There are many possibilities for their usage, especially in medicine, pharmacy and the food industry. For instance, seaweeds have been utilized industrially as a source of agar, carrageenans and alginates [1,10,11] and freshwater algae and seaweeds have been evaluated as nutraceutical foods [12,13].

Lipids, including their fatty acids (FAs), are essential human nutrients that can be classified as saturated (SFAs), monounsaturated (MUFAs), and polyunsaturated FAs (PUFAs), according to the absence or presence of unsaturated bonds. Humans are able to synthesize SFAs and MUFAs, but unfortunately, PUFAs with the first double bond on the third or sixth carbon atom (essential fatty acids—EFAs) are essential because they cannot be synthesized by humans [4,14].

Generally, the total lipid content of seaweeds by dry weight varies from 1% to 6% [15,16]. On the other hand, the total lipid content of freshwater green microalga *Chlorella* sp. was in the range from 2% to 22% by dry weight and the total lipid content of *Spirulina* sp. cyanobacterium ranged from 6.4% to 14.3% by dry weight [17,18,19]. Despite a low lipid content, seaweeds are rich in ω-3 and ω-6 FAs [15,16,20]. Red and brown seaweeds have a high concentration of eicosapentaenoic acid (EPA, C20:5) and arachidonic acid (C20:4), green algae are rich in α-linolenic acid (ALA, C18:3) and *Spirulina* cyanobacteria are rich in γ-linolenic acid (GLA, C18:3) [21,22].

The contemporary Western human diet is known for an increased intake of SFAs and ω-6 FAs that results in an imbalance of ω-3 and ω-6 FAs [4,23]. Seaweeds contain a higher proportion of ω-3 FAs, which are components of all cell membranes and are precursors of biochemical and physiological reactions in the body, acting against atherosclerosis, hypertension, inflammatory diseases, cystic fibrosis, rheumatoid arthritis and helping prevent mental illnesses [2,4,24].

The chemical composition of seaweed is affected by many factors such as the seaweed species, location and time of harvest, intensity of light, water chemistry at the location, and the part of plants used [2,25]. Studies of the lipid profile in seaweeds have investigated the seasonal variation [26], effect of growth conditions [27,28], and differences among diverse seaweed tissues [29]. Most studies which were focused on the total lipid contents and on the FA profiles have concerned fresh seaweeds [26,28,29]. However, the effect of algae and seaweed processing, *i.e.*, drying, packaging, transporting, and subsequent storage, on the lipid content and FAs composition is rarely studied.

The present paper evaluates and compares the lipid profiles of nine commercially available seaweed microalgal and cyanobacterial products, and autotrophically cultivated samples of the green microalga *Chlorella kessleri* and the cyanobacterium *Spirulina platensis*. In addition, yield of lipids and FA profiles of seaweeds extracted using different solvents were compared. The new information presented herein should be useful to support more abundant consumption of dry seaweed products as a source of ω-3 and ω-6 FAs, and also for better revaluating the real contribution of freshwater algal and seaweed products to PUFA enrichment of the human food chain.

## 2. Results and Discussion

### 2.1. Total Lipid Contents

Total lipid contents of analyzed samples were determined in extracts obtained by different solvents (Table 1). Evidently, extraction using a 1:2:1 mixture of methanol/chloroform/water (solvent I) resulted in higher contents of lipids among all determined samples, ranging from 1.32% (*P. palmata*) to 18.02% (*S. platensi*s), whereas the extraction with hexane (solvent II) was less effective in relation to lipid contents, that ranged from 0.64% (*P. palmata*) to 13.41% (*S. platensis*) for the same algal samples as in the previous analysis.

Moreover, the total lipid content seemed to be influenced by the cultivation or technology process. Here, the results showed much higher total lipid contents in autotrophically cultivated microalgae compared to freshwater edible products in pill form using both solvent systems. In detail, autotrophically cultivated cyanobacterium *S. platensis* contained 18.02% lipids (I), in comparison with 10.23% in the spirulina product (I). Similarly, 18.01% was measured in the autotrophically cultivated green microalga *C. kessleri* compared to 3.70% in the green microalga *C. pyrenoidosa* product after extraction with solvent I. Further, the content of total lipids obtained from the green microalga *C. pyrenoidosa* with solvent I (3.70%) was lower than established by Ortega-Calvo *et al.* in dry *C. vulgaris* algal product (8.6%) after extraction by dichloromethane/methanol (2:1) [19]. Similarly, D’Oca *et al.* presented results of lipid contents in *C. pyrenoidosa* in the range from 1.55% to 20.74%, depending on the different solvents and extraction methods used [18].

**Table 1:** Total lipid content (%) of dry samples of cyanobacterial, microalgal and seaweed products and autotrophically cultivated *S. platensis* and *C. kessleri*, in means ± SD.

Sample ^†^	Total lipid content [%]			
	Mean ^1^	SD ^1^	Mean ^2^	SD ^2^
S	10.23 ^a^	0.59	3.50 ^b^	0.37
SP	18.02 ^a^	1.15	13.41 ^b^	0.79
C	3.70 ^a^	0.31	3.41 ^a^	0.32
CK	18.01 ^a^	1.15	9.87 ^b^	0.89
N	1.61 ^a^	0.10	0.93 ^b^	0.13
D	1.32 ^a^	0.18	0.64 ^b^	0.10
K	3.11 ^a^	0.26	1.83 ^b^	0.19
A	2.02 ^a^	0.22	1.58 ^b^	0.12
W	1.66 ^a^	0.15	0.73 ^b^	0.17
WI	3.31 ^a^	0.34	1.11 ^b^	0.15
H	1.55 ^a^	0.16	1.17 ^b^	0.08

**^†^** *Spirulina platensis*—S; *Spirulina platensis* cultivated autotrophically in a solar photobioreactor—SP; *Chlorella pyrenoidosa—*C; *Chlorella kessleri* cultivated autotrophically in a solar photobioreactor—CK; *Porphyra tenera*—N; *Palmaria palmata*—D; *Laminaria japonica*—K; *Eisenia bicyclis*—A; *Undaria pinnatifida*—W, WI; *Hizikia fusiformis*—H; ^1^ extraction with a 1:2:1 solvent mixture of methanol/chloroform/water (solvent I); ^2^ extraction with hexane (solvent II); ^a,b^ values in a row without a common superscript are significantly different (*p* < 0.05).

A similar result (14.3%) for total lipid content in the cyanobacterium *S. platensis* after extraction by a mixture of chloroform/methanol (2:1) was published by Babadzhanov *et al.* [17]. On the other hand, Ortega-Calvo *et al.* reported much lower contents of lipids in the cyanobacterial food products of *S. platensis*, *S. maxima*, and *Spirulina* sp. (6.4%–7.5% in dry weight) using a mixture of dichloromethane/methanol (2:1) [19].

The total lipid contents after extraction of the red seaweed products by solvent I were in the range from 1.32% (*P. palmata*) to 1.61% (*P. tenera*). These results are in accordance with literature lipid values in *Porphyra* sp. (1.0%–2.8%) and *P. palmata* (0.9%–1.8%) [15,19,20], although those lipid extractions were effected using different solvent mixtures: methanol/chloroform/water (1:2:1) [15], dichloromethane/methanol (2:1) [19] and chloroform/methanol (2:1) [20,30].

Lipid contents after extraction of brown algal products with solvent I were in the range from 1.55% (*H. fusiformis*) to 3.31% (*U. pinnatifida* WI). Observed total lipids results in brown seaweeds were largely in accordance with reported values: *L. japonica* 1.0% [15], *Eisenia arborea* 0.6% [31], *U. pinnatifida* 1.05%–4.5% [15,20], and finally *H. fusiformis* 0.7%–1.4% [15,19]. However, depending on the variability of the chemical composition of algae and methods of analysis, reported results are highly variable.

It was obvious that freshwater green microalgae and cyanobacteria contained higher concentrations of lipids than seaweed products. This could be caused by the specific metabolism and growth conditions of these algae.

In accordance with our findings, significant discrepancies in the efficiency of various solvent mixtures used for lipid extraction were reported in several studies [15,17,18,19,20]. That could be caused by the presence of different lipid components in the algal biomass. Generally, a higher amount of polar lipid compounds in algal biomass results in worse lipid extraction yields by nonpolar solvents and *vice versa* [18].

### 2.2. Influence of Extractive Solvents on the Number and Amount of Identified Fatty Acids

**Figure 1:**
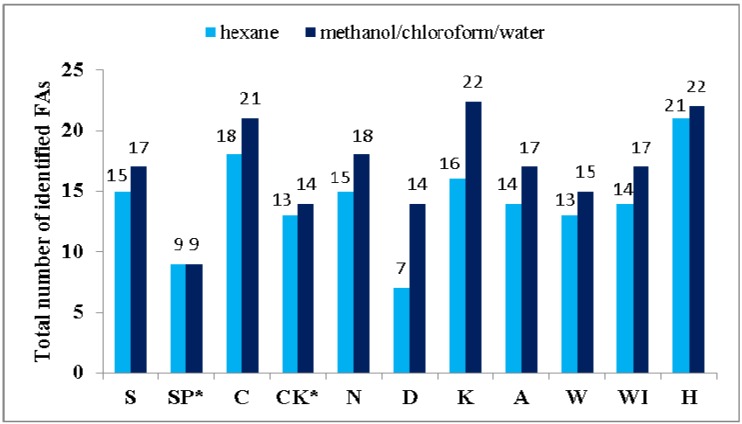
Numbers of identified FAs in cyanobacterial, microalgal and seaweed products and autotrophically cultivated *S. platensis* and *C. kessleri* extracted by solvent I or II.

**Figure 2:**
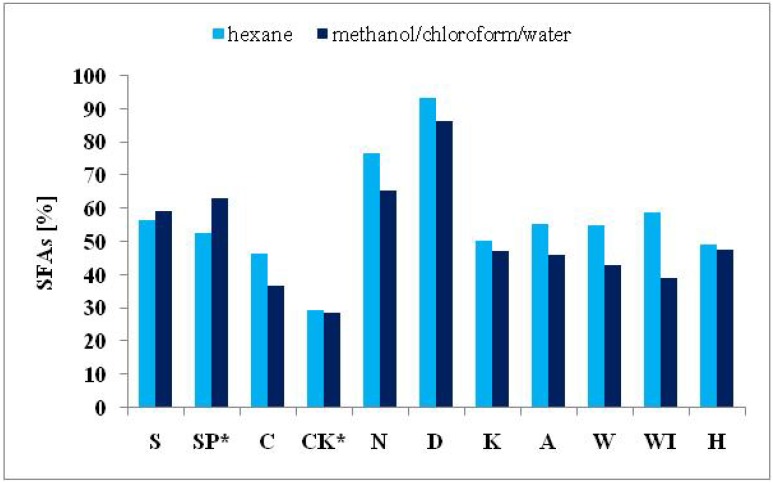
Comparison of the SFA contents (expressed in % of total FAMEs) extracted from cyanobacterial, microalgal and seaweed products and autotrophically cultivated *S. platensis* and *C. kessleri* by solvent I and II.

**Figure 3:**
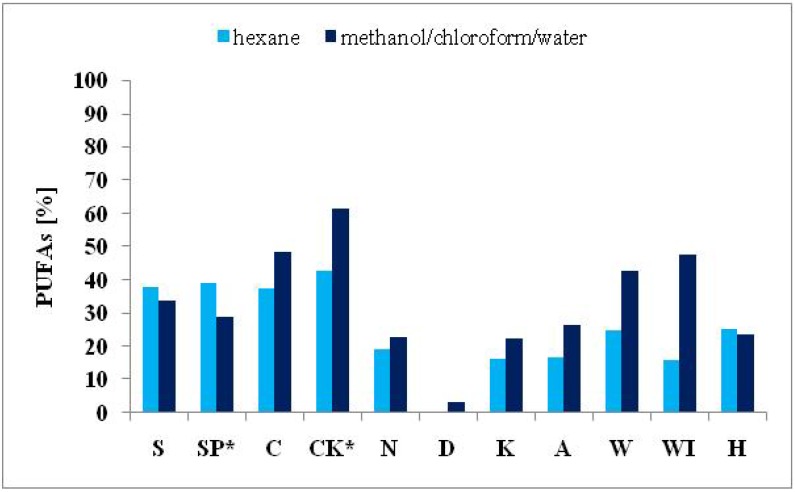
Comparison of the PUFA contents (expressed in % of total FAMEs) extracted from cyanobacterial, microalgal and seaweed products and autotrophically cultivated *S. platensis* and *C. kessleri* by solvent I and II.

**Figure 4:**
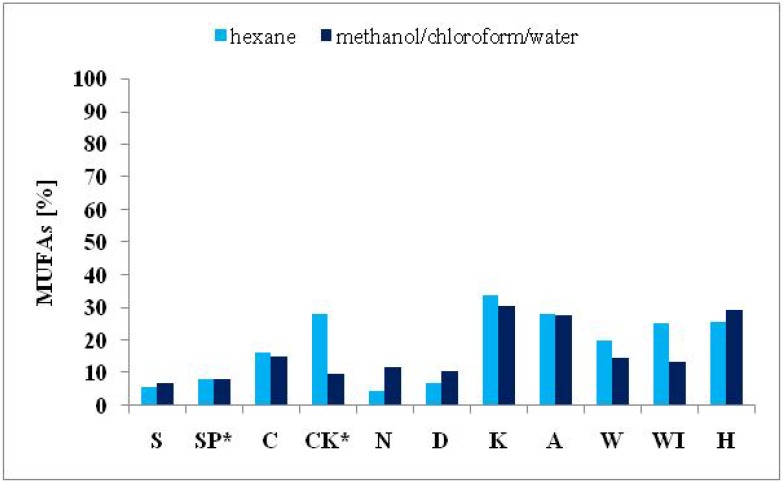
Comparison of the MUFA contents (expressed in % of total FAMEs) extracted from cyanobacterial, microalgal and seaweed products and autotrophically cultivated *S. platensis* and *C. kessleri* by solvent I and II.

Significant differences in the FA composition of the nine algal products and the autotrophically cultivated *S. platensis* and *C. kessleri* were established. Variations in FA contents are ordinarily attributable both to environmental and genetic differences [20,32]. Nevertheless, the extractive solvents had an influence on the number and amount of identified FAs. The impact of the solvents used on the total number of identified FAs and different amounts of determined SFAs, MUFAs and PUFAs are presented in Figure 1, Figure 2, Figure 3 and Figure 4.

From Figure 1 can be concluded that 9 to 22 or 7 to 21 FAs were identified, respectively, depending on whether solvent I or II was used. The solvent I was found more effective than solvent II in relation to a number of identified FAs for almost all samples, except for autotrophically cultivated *S. platensis*, where the same efficiency for both solvents was established.

The different efficiency of the two solvents used for the extraction of algal lipids in relation to the proportions of the different FAs is evident too from the obtained results shown in Figure 2, Figure 3 and Figure 4. Higher amounts of SFAs (Figure 2) were obtained from all samples except for *Spirulina* genus using solvent II. The solvent I was more effective for PUFAs for almost in all samples, except for both *Spirulina* genus samples and the product from the brown seaweed *H. fusiformis* (Figure 3). Further, solvent II was more effective for MUFA extraction in both samples of the green freshwater algae *C. kessleri* and *C. pyrenoidosa* and in the three brown seaweed products *L. japonica*, and *U. pinnatifida* (W, WI). Finally, in samples of autotrophically cultivated *S. platensis* and *E. bicyclis*, no difference between the two solvents used was observed (Figure 4).

### 2.3. Fatty Acid Profiles

FA compositions of cyanobacterial, microalgal and seaweeds products and two samples of autotrophically cultivated green microalga and cyanobacteria obtained by different solvent extractions are presented in Table 2 and Table 3 and the results are given in % of total FAMEs.

**Table 2:** FA profiles of the cyanobacterial, microalgal and seaweed products and autotrophically cultivated cyanobacterium *S. platensis* and microalga *C. kessleri* (% of total FAMEs) of lipid extracted by solvent I.

	Cyanobacteria		Green microalgae		Red seaweeds		Brown seaweeds				
FAs	S	SP *	C	CK *	N	D	K	A	W	WI	H
C10:0	0.11	nd	0.20	nd	1.40	0.57	0.04	0.13	0.10	0.09	0.16
C12:0	0.21	nd	0.08	0.11	0.29	0.58	0.05	0.08	0.10	0.11	0.13
C14:0	0.56	0.46	0.44	1.14	1.99	12.32	5.82	8.18	5.88	5.16	5.09
C15:0	0.10	nd	0.12	0.72	0.88	1.79	0.20	0.59	0.44	0.33	0.45
C16:0	53.02	61.06	25.59	24.64	57.61	65.49	31.21	31.10	33.38	31.05	33.80
C17:0	0.28	0.27	0.36	0.29	0.28	0.45	0.20	0.28	0.36	0.34	0.39
C18:0	4.75	1.40	9.48	0.74	1.40	3.45	5.00	0.96	2.04	1.43	1.19
C20:0	0.06	nd	0.09	nd	nd	nd	0.67	1.36	0.78	0.65	0.19
C21:0	nd	nd	nd	nd	0.36	nd	nd	nd	nd	nd	0.92
C22:0	nd	nd	0.16	nd	1.71	nd	nd	nd	nd	nd	0.49
C24:0	0.38	nd	0.47	1.23	nd	1.95	4.06	3.42	nd	nd	4.68
C16:1(n-7)	3.36	2.59	1.49	1.61	2.20	5.01	0.79	10.04	1.80	1.04	5.25
C17:1(n-7)	0.50	nd	0.15	nd	0.42	nd	0.09	0.57	nd	0.13	0.44
C18:1trans (n-9)	0.08	nd	5.05	2.50	1.49	nd	0.20	nd	nd	nd	nd
C18:1cis (n-9)	2.79	5.29	8.06	5.50	3.79	5.29	26.45	13.39	13.32	11.91	10.00
C20:1(n-9)	nd	nd	nd	nd	3.98	nd	2.83	3.54	nd	nd	9.86
C22:1(n-9)	nd	nd	nd	nd	nd	nd	nd	nd	nd	0.22	3.51
C18:2cis (n-6)	16.17	18.42	18.79	36.38	8.90	0.71	8.76	9.55	10.12	9.85	5.07
C18:3(n-3)	nd	nd	21.29	19.76	3.52	0.38	1.22	nd	10.48	17.17	1.46
C18:3(n-6)	17.36	10.44	7.64	4.74	8.66	1.96	1.94	3.02	5.74	7.52	2.43
C20:2(n-6)	0.11	nd	0.26	0.64	nd	nd	0.93	nd	nd	nd	nd
C20:3(n-6)	0.15	nd	0.14	nd	nd	nd	0.94	0.34	0.79	0.61	0.35
C20:4(n-6)	nd	nd	nd	nd	1.48	nd	8.57	13.54	15.37	12.38	13.97
Other ^a^	0.00	0.07	0.13	0.00	0.00	0.08	0.05	0.00	0.00	0.00	0.16
ΣSFAs	59.47	63.18	36.99	28.87	65.56	86.58	47.24	46.01	43.09	39.17	47.49
ΣMUFAs	6.73	7.96	14.78	9.61	11.88	10.38	30.38	27.54	14.40	13.30	29.07
ΣPUFAs	33.79	28.86	48.23	61.52	22.56	3.04	22.39	26.45	42.51	47.53	23.44
Σ_n3-FAs_	0.00	0.00	21.29	19.76	3.52	0.38	1.22	0.00	10.48	17.17	1.46
Σ_n6-FAs_	33.79	28.86	26.93	41.42	19.04	2.67	21.17	26.45	32.02	30.36	21.98

* samples cultivated autotrophically in a solar photobioreactor; nd—FAs not detected; Other ^a^ includes FAs determined in trace amounts, such as C14:1(n-5), C24:1(n-9), C18:2*trans*(n-6).

**Table 3:** FA profiles of the cyanobacterial, microalgal and seaweed products and autotrophically cultivated cyanobacterium *S. platensis* and microalga *C. kessleri* (% of total FAMEs) of lipid extracted by solvent II.

	Cyanobacteria		Green microalgae		Red seaweeds		Brown seaweeds				
FAs	S	SP *	C	CK *	N	D	K	A	W	WI	H
C10:0	0.19	nd	0.09	nd	0.58	3.73	0.16	0.42	0.71	0.84	0.30
C12:0	0.36	0.10	0.17	0.47	0.96	2.45	nd	nd	0.29	0.60	0.17
C14:0	0.71	0.48	0.57	2.25	1.97	13.16	6.67	9.56	6.64	7.47	5.49
C15:0	0.12	nd	0.13	1.08	3.24	nd	0.13	0.71	nd	0.78	0.50
C16:0	44.85	50.43	27.53	20.33	46.50	48.82	34.67	38.35	35.49	36.93	34.36
C17:0	0.43	0.51	0.39	1.37	3.81	13.17	0.32	1.04	1.85	0.42	0.54
C18:0	9.90	1.30	16.83	3.79	7.58	11.93	5.55	2.97	8.61	6.71	1.31
C20:0	0.12	nd	0.16	nd	3.23	nd	0.71	1.50	1.42	0.57	0.40
C21:0	nd	nd	0.03	nd	nd	nd	nd	0.91	nd	nd	0.99
C22:0	nd	nd	nd	nd	nd	nd	nd	nd	nd	nd	1.36
C24:0	nd	nd	0.65	nd	8.70	nd	2.20	nd	nd	4.53	3.91
C16:1(n-7)	2.56	3.10	1.52	1.36	1.28	nd	0.75	9.44	2.76	1.10	2.67
C17:1(n-7)	0.42	nd	0.10	nd	0.21	nd	nd	nd	nd	nd	0.33
C18:1trans (n-9)	0.08	nd	3.44	1.91	0.04	nd	0.20	nd	nd	nd	nd
C18:1cis (n-9)	2.46	5.13	11.00	7.86	2.87	6.74	30.49	16.28	9.35	15.30	11.28
C20:1(n-9)	nd	nd	nd	nd	nd	nd	2.11	2.11	7.95	8.85	8.46
C22:1(n-9)	nd	nd	nd	nd	nd	nd	nd	nd	nd	nd	2.78
C18:2cis (n-6)	13.65	23.73	16.12	25.89	8.44	nd	8.30	7.94	4.92	9.88	5.60
C18:3(n-3)	nd	nd	13.41	10.86	nd	nd	nd	nd	nd	nd	1.97
C18:3(n-6)	23.98	15.22	7.78	5.95	10.60	nd	1.71	2.47	19.16	nd	2.28
C20:2(n-6)	0.16	nd	0.08	nd	nd	nd	nd	nd	nd	nd	nd
C20:3(n-6)	nd	nd	nd	nd	nd	nd	0.62	nd	nd	nd	0.91
C20:4(n-6)	nd	nd	nd	nd	nd	nd	5.41	6.29	0.85	6.03	14.39
Other ^a^	0.00	0.00	0.00	16.88	0.00	0.00	0.00	0.00	0.00	0.00	0.00
ΣSFAs	56.70	52.82	46.56	29.29	76.56	93.26	50.41	55.46	55.02	58.84	49.34
ΣMUFAs	5.51	8.23	16.06	28.01	4.41	6.74	33.55	27.84	20.05	25.24	25.52
ΣPUFAs	37.79	38.95	37.38	42.70	19.04	0.00	16.03	16.70	24.93	15.91	25.15
Σ_n3-FAs_	0.00	0.00	13.41	10.86	0.00	0.00	0.00	0.00	0.00	0.00	1.97
Σ_n6-FAs_	37.79	38.95	23.97	31.84	19.04	0.00	16.03	16.70	24.93	15.91	23.18

* Samples cultivated autotrophically in a solar photobioreactor; nd—FAs not detected, Other ^a^ includes FAs determined in trace amounts, such as C24:1(n-9).

#### 2.3.1. Saturated Fatty Acids

Pursuant to published data, the most abundant groups of algal lipids among the total FAMEs are SFAs or PUFAs, depending on the algal species [15,33]. The majority of the investigated samples showed the highest proportions of SFAs in their FAMEs distribution regardless of the solvent used. The highest contents of SFAs obtained with the solvents I and II were established in the red seaweeds *P. palmata* (86.58%/93.26%) and *P. tenera* (65.56%/76.56%). Conversely, the cultivated freshwater green microalga *C. kessleri* had the lowest contents of SFAs (28.87%/29.29%).

Palmitic acid (C16:0) was found to be the predominant SFA in all samples, present in a range from 24.64% of total FAMEs (cultivated *C. kessleri*, I) to 65.49% (*P. palmata*, I) and from 20.33% (cultivated *C. kessleri*, II) to 50.43% (cultivated *S. platensis*, II).

Comparable contents of palmitic acid (C16:0) were found in the cyanobacterial *S. platensis* product (53.02%/44.85%) and in autotrophically cultivated *S. platensis* (61.06%/50.43%) in comparison with the reported amounts (49.1%–56.2%) for *Spirulina* sp., *S. platensis* and *S. maxima* [19], and for autotrophically cultivated *S. platensis* (44.9%) [17].

Likewise, comparable contents of palmitic acid (C16:0) were established in the green microalgae *C. pyrenoidosa* (25.59%/27.53%) and in cultivated *C. kessleri* (24.64%/20.33%) compared to reported data for the microalgal product *C. vulgaris* (22%, 25.1% of total FAMEs) [19,34].

Contents of palmitic acid (C16:0) in the red seaweeds *P. palmata* (65.49%/48.82%) and *P. tenera* (57.61%/46.50%) were determined as the highest from almost all samples with both solvents. These results are in accordance with the published data for *Porphyra* sp. (30.8%–63.19%) [15,20] and *P. palmata* (23.3%–65.8%) [19,35].

Generally, contents of palmitic acid (C16:0) in brown seaweed products obtained by solvent II (34.36%–38.35%) were surprisingly higher than those obtained by solvent I (31.10%–33.80%). It was observed that the content of palmitic acid (C16:0) in *L. japonica* (31.21%/34.67%) was in keeping with the reported content of palmitic acid (C16:0) in the brown seaweed *Laminaria* sp. (36.0%) [15], but differed from the result reported for the same seaweed *L. japonica* (12.3%) [36]. Further, in *H. fusiformis*, higher amounts of palmitic acid (33.80%/34.36%) were found in comparison with the reported contents (26.8%–31.0%) [15,19]. Similarly, in the samples of the other brown seaweed *U. pinnatifida* (W, WI), higher amounts of palmitic acid (C16:0) were measured (33.38%/35.49% for W; 31.05%/36.93% for WI) in comparison with the published data (13.5%–26.8%) [15,36]. SFAs with a higher carbon number (C_20_–C_22_) mostly occurred in small amounts or they were not identified in the analyzed samples, except for C_24_ in both extracts.

#### 2.3.2. Monounsaturated Fatty Acids

MUFAs were distributed in less amounts than SFAs and their contents ranged from 6.73% (*S. platensis*) to 30.38% (*L. japonica*) in the solvent I extract and from 4.41% (*P. tenera*) to 33.55% (*L. japonica*) in solvent II extracts. In general, large differences were found in MUFA contents among the analyzed algal species. The highest contents of MUFAs were determined in brown seaweeds, whilst the lowest contents were detected in the samples of cyanobacteria and red seaweeds, depending on the solvents used.

Oleic acid C18:1(n-9) was found as the most abundant MUFA in all samples, except for *S. platensis* and *P. tenera*, where palmitooleic acid C16:1(n-7) and eicosenoic acid C20:1(n-9), were established as the predominant MUFAs, respectively. Contents of oleic acid expressed as a sum of C18:1(n-9) ranged from 2.86%/2.54% (*S. platensis*) to 26.65%/30.69% (*L. japonica*) of total FAMEs. However, the results of oleic acid C18:1(n-9) determined in almost all samples differed from the values reported in literature (e.g., oleic acid in *Spirulina* sp., *S. platensis*, and *S. maxima* in trace amounts [19] *versus* 10.1% in autotrophically cultivated *S. platensis* [17]).

The values (8.06%/11.00%) of oleic acid (C18:1) in *C. pyrenoidosa* also did not match the reported value (5%) in photoautotrophically cultivated *C. pyrenoidosa* [34], or even in a microalgal product of another genus, *C. vulgaris* (25.3%) [19].

Further, disagreement with published data concerning oleic acid in red seaweed products was established. The determined sums of oleic acid C18:1(n-9) in *P. tenera* (5.29%/6.74%) were lower compared to reported data for *Porphyra* sp*.* (7.16%–15.3% of total FAMEs expressed as a sum of C18:1 [15]; 6.70% of oleic acid C18:1(n-9) of total FAMEs [20]). However, a higher content (5.29%/6.74%) as the sum of oleic acids C18:1(n-9) was analyzed in the other red seaweed *P. palmata* contrary to the published value for *Palmaria* sp. (3.13%) [20].

In keeping with reports on brown seaweeds, MUFAs with higher numbers of carbons were identified as more abundant than in other algal species. The summed contents of oleic acid C18:1(n-9) in *L. japonica* gave the highest amount (26.65%/30.69%) of total FAMEs, unlike reported data (8.4%) [36]. In the two products from *U. pinnatifida* (W, WI), higher contents of oleic acid C18:1(n-9) (13.32%/9.35% in W; 11.91%/15.30% in WI) were determined than reported in published data (6.79%–10.2%) [20,35]. The same situation was observed in the last product from the brown seaweed *H. fusiformis*, where 10.00% and 11.28% were determined, contrary to a published 7.68% values for total FAMEs expressed as a sum of C18:1 [15].

Eicosenoic acid C20:1(n-9) was identified in the red seaweed *P. tenera* (3.98%), in three products from brown seaweeds (2.83% in *L. japonica*, 3.54% in *E. bicyclis* and 9.86% in *H. fusiformis*, all extracted with solvent I). These data differed from published values (1.42%, 1.52% in *P. tenera* [15]; 4.70% in *Porphyra* sp. [20]; 0.2%, 0.6% in *P. palmata* [20,35]; 1.55% in *Laminaria* sp.; 4.09% in *H. fusiformis* [15]). In solvent II extracts, eicosenoic acid C20:1(n-9) was only identified in products from brown seaweeds (2.11%–8.85%).

#### 2.3.3. Polyunsaturated Fatty Acids

PUFA contents ranged from 3.04% (*P. palmata*) to 61.52% (cultivated *C. kessleri*) using solvent I, whereas solvent II extracts were in the range from 0.00% (*P. palmata*) to 42.70% (cultivated *C. kessleri*) of total FAMEs. The extraction of lipids by solvent II seemed to be insufficient for the isolation of PUFAs with higher carbon numbers, as they were not detected in most of the analyzed samples, except for C20:4(n-6) determined in the brown seaweed products.

Predominantly, PUFAs were the most abundant; namely linoleic acid C18:2(n-6) which varied from 0.71%/0.00% (*P. palmata*) to 36.38%/25.89% (cultivated *C. kessleri*) of total FAMEs; α-linolenic acid C18:3(n-3) which ranged from 0.00% (*S. platensis*, cultivated *S. platensis*, *Eisenia bicyclis*) to 21.29% (*C. pyrenoidosa*); γ-linolenic acid C18:3(n-6) in the range from 1.94% (*L. japonica*) to 17.36% (*S. platensis*) (all with solvent I) and in solvent II extracts from 0.00% (*P. palmata*, *U. pinnatifida* WI) to 23.98% (*S. platensis*). Finally, in three products from brown seaweeds: *E. bicyclis*, *U. pinnatifida* (W), and *H. fusiformis*, arachidonic acid C20:4(n-6) was the most represented PUFA (13.54%–15.37%, I; 0.85%–14.39%, II).

The content of linoleic acid (C18:2) in the analyzed *S. platensis* was approximately the same as reported for *Spirulina* sp., *S. platensis* and *S. maxima* (12.6%–21.7%) [19] and *S. platensis* (11.1%) [17]. However, C18:3(n-3) was not identified in the surveyed samples, compared to the published values of C18:3 in *Spirulina* sp., *S. platensis*, and *S. maxima* (5.9%–15.8%) [19] and in *S. platensis* (17.1%) [17].

According to Petkov and Garcia [34], linoleic acid (C18:2) was present in *C. vulgaris* in a higher amount (24.0%) than in the analyzed sample of *Chlorella pyrenoidosa* (18.79%, I), whilst the reported amount (18.0%) in photoautotrophically cultivated *C. pyrenoidosa* was half that in the analyzed sample of autotrophically cultivated *C. kessleri* (36.38%, I). On the other hand, an agreement with the C18:3(n-3, 6, 9) result, 27% was measured in a photoautotrophically cultivated *C. pyrenoidosa* [34] compared with the analyzed samples.

In the samples from red seaweeds, higher amounts of linoleic acid C18:2(n-6) were established in *P. tenera* than published for *Porphyra* sp. (1.17%–7.06%) [15,20]. Further, agreement with published C18:2(n-6) content (0.3%) in *P. palmata* was found [35].

The observed content of linoleic acid C18:2(n-6) in the product from the brown seaweed *L. japonica* was in agreement with data reported for the same species (8.4%) [36], while a lower amount (6.79%) of this PUFA was found in another genus, *Laminaria ochroleuca* [20]. A lower amount of linoleic acid C18:2(n-6) (6.23%) was reported for another brown *U. pinnatifida* seaweed [20] than determined in our analyzed samples from *U. pinnatifida* (W, WI). With respect to the presentation of other important PUFAs in the products from *U. pinnatifida*, similar results to those reported for α-linolenic acid C18:3(n-3) (5.8%–11.97%) [20,37], and for arachidonic acid C20:4(n-6) (12.7%–17.5%) [35,36] were found. Finally, in the product from *H. fusiformis*, agreement with the amount of arachidonic acid C20:4(n-6) with the published data (14.1%) was observed [19], while less content (5.30%) of this PUFA was reported by other authors [15].

Generally, α-linolenic (ALA) and linoleic acids (LA) are the primary precursors of ω-3 and ω-6 EFAs, respectively. Both are formed by the gradual desaturation of oleic acid in the endoplasmic reticulum and plantae chloroplasts. Importantly, humans cannot synthesize ALA due to the absence of the ∆^12^ and ∆^15^ desaturases required for the synthesis of ALA from stearic acid (18:0) or PUFAs with the first double bond on the C3 (ω-3) and C6 (ω-6) from the methyl-end. Thus, the level of these PUFAs in the human body depends on their intake from the diet [4]. Generally, ω-3 PUFAs play crucial roles in many biochemical pathways which results in various health benefits, especially cardioprotective effects that result from their considerable anthiatherogenic, antithrombotic, anti-inflammatory, antiarrhytmic, hypolipidemic effects, and other health benefits, based on the complex influence of the concentrations of lipoproteins, fluidity of biological membranes, function of membraned enzymes and receptors, modulation of eicosanoids production, blood pressure regulation, and finally on the metabolism of minerals [4,38,39,40,41,42].

Fish oil is considered as the main source of essential PUFAs. Nevertheless, fish also cannot synthesize these PUFAs because of the absence of crucial enzymes and the high level of essential PUFAs in fish oil is a direct consequence of the presence of marine microorganisms and algae in the fish trophic chain. The highest content of ω-3 FAs was determined in the green microalga *C. pyrenoidosa* (21.29%, I) and the highest content of ω-6 FAs was observed in the other cultivated freshwater green microalga *C. kessleri* sample (41.42%, I).

### 2.4. Fatty Acid Profiles of Autotrophically Cultivated Cyanobacteria and Microalga

Autotrophically cultivated *S. platensis* showed a similar FA composition to the cyanobacterial product of *S. platensis*, except for a slightly higher content of C16:0 and lower content of C18:3(n-6) in the cultivated alga. In contrast, the other autotrophically cultivated freshwater green alga *C. kessleri* showed a higher amount of PUFAs than the microalgal product of *C. pyrenoidosa*, the highest of all analyzed samples. The content of linoleic acid C18:2(n-6), which was the predominant PUFA in cultivated *C. kessleri*, exceeded the amount of this PUFA in product from *C. pyrenoidosa* by 51.6%.

### 2.5. PUFAs/SFAs Fatty Acids Ratio

The PUFAs/SFAs fatty acids ratio (hereinafter referred to as ratio) could be used for a rapid evaluation of FA profiles of analyzed samples; the higher value of this ratio means more health benefits. Ratio in the product from *S. platensis* (0.57/0.66) and in autotrophically cultivated *S. platensis* (0.46/0.74) are in accordance with the reported ratios in *Spirulina* sp., *S. platensis*, and *S. maxima* (0.25–0.75) [19] and in the cyanobacterium *S. platensis* (0.54) [17].

The determined ratios (1.30/0.80) of the *C. pyrenoidosa* product was slightly higher than reported (0.71) for the microalgal product from *C. vulgaris* [19]. On the other hand, the ratios (2.13/1.46) of autotrophically cultivated *C. kessleri* were fractionally lower than the ratio (2.83) of photoautotrophically cultivated *C. pyrenoidosa* [34].

In the red seaweed products of *P. tenera* (0.34/0.06) and *P. palmata* (0.04/0.07), the lowest ratios of all samples due were measured to the highest levels of SFAs. Several authors have also reported low ratios in different red seaweed species, such as *Porphyra* sp. (0.76, 1.25 [15]; 0.25 [20]) and *P. palmata* (0.0–0.48 [19,20]).

Established low values of ratios in the products from the brown seaweeds *L. japonica* (0.47/0.32), *E. bicyclis* (0.57/0.30), *U. pinnatifida* (W, 0.99/0.45; WI, 1.21/0.27), and *H. fusiformis* (0.49/0.51) agreed with a higher presence of SFAs in their lipids, except for the two products from *U. pinnatifida* (W, WI), where an equal or higher amount of PUFAs were determined in the extracts obtained with solvent I. Higher ratios for *Laminaria* sp. (0.96), *U. pinnatifida* (4.21) and *H. fusiformis* (2.02) [15] and similar ratio values in *U. pinnatifida* (1.10) and in *H. fusiformis* (0.5) [19] were previously published.

Based on the obtained results and data presented in literature [15,17,18,19,20], it is evident very significant differences exist within the FA profiles in the same species of algae and seaweeds depending on the used solvents and methods of analysis. Further, chemical composition of seaweed and microalgae is affected by many factors (species of seaweed, location and time of harvest, light intensity, water chemistry and the used part of plants); therefore, the results obtained from various analyses may differ [2,25].

## 3. Experimental

### 3.1. Samples and Chemicals

The study was conducted with eight representative species of dried cyanobacterial, microalgal and seaweed products purchased in a special local store in dried form; they were represented by green microalga (*Chlorophyta*), cyanobacteria (*Cyanophyceae*), brown seaweeds (*Phaeophyta*), red seaweeds (*Rhodophyta*), and two samples of autotrophically cultivated freshwater green microalga *Chlorella kessleri* (No. 260) and cyanobacterium *Spirulina platensis* (No. 27) obtained from the Culture Collection of Autotrophic Organisms, Institute of Botany, Academy of Sciences of the Czech Republic, Centre of Phycology (Trebon, Czech Republic). Both autotrophically cultivated species were harvested in exponential growth phase. Characteristics of all the samples are summarized in Table 4.

**Table 4:** Characteristics of cyanobacterial, microalgal and seaweed samples.

Algal group	Algal species	Sample	Product	Country	Form
Green	*Chlorella pyrenoidosa*	C	Chlorella Tabs	Taiwan	pills
	*Chlorella kessleri*	CK *	-	Czech Republic	dried alga
Cyanobacteria	*Spirulina platensis*	S	Spirulina Bio	India	pills
	*Spirulina platensis*	SP *	-	Czech Republic	dried alga
Red	*Porphyra tenera*	N	Nori flakes	Japan	dried seaweed
	*Palmaria palmata*	D	Dulse flakes Bio	USA	dried seaweed
Brown	*Laminaria japonica*	K	Kombu	Japan	dried seaweed
	*Eisenia bicyclis*	A	Arame	Japan	dried seaweed
	*Undaria pinnatifida*	W	Wakame	Japan	dried seaweed
	*Undaria pinnatifida*	WI	Wakame-instant	Japan	dried seaweed
	*Hizikia fusiformis*	H	Hijiky	Japan	dried seaweed

* samples cultivated autotrophically in a solar photobioreactor.

All product samples were pulverized with a mixer (Vorwerk Thermomix TM 31, Wuppertal, Germany) to obtain a homogenous powder with a particle size of 1 mm and they were stored in airtight plastic bags at room temperature (25 °C). Freshwater green microalga *Chlorella kessleri* and cyanobacterium *Spirulina platensis* were cultivated autotrophically in a solar photobioreactor as described in the study by Masojídek *et al.* [43]. For the cultivation of microalgae, BG11 culture medium was used [44]. After the cultivation, the algal biomass was lyophilized (Alpha 1-4 LSC, Christ, Osterode am Harz, Germany) and stored in airtight plastic bags at room temperature (25 °C). All used chemicals were of analytical grade and were purchased from Merck (Darmstadt, Germany), except for the standard mixture of 37 FAMEs (FAME Mix, Supelco, Bellefonte, PA, USA), and methyl undecanoate purchased from Sigma Aldrich Chemical Co. (St. Louis, MO, USA).

### 3.2. Total Lipids Determination

Total lipids of the analyzed samples were extracted using two different solvents. Either a mixture of methanol/chloroform/water (1:2:1, v/v/v) according to the modified method of [45] or *n*-hexane was used. Specifically, a portion (2 g) of every dried ground sample was weighed into an extraction thimble and subjected to a Soxhlet extraction for 4 h with 100 mL of the solvent mixture. Subsequently, the solvent was removed on a vacuum rotary evaporator (Laborota 4010 Digital, Heidolph, Schwabach, Germany) and the lipid extracts were dried at 105 °C for 2 h (Venticell 111 Komfort, BMT, Brno, Czech Republic). The amount of total lipid contents of all samples was determined gravimetrically [18].

### 3.3. GC Analysis of FAMEs

FAs were determined by gas chromatography (GC) of their methyl esters (FAMEs) in the lipid extracts obtained by above described method, excluding drying. Briefly, 0.5 M sodium hydroxide in methanol (4 mL) was added to the lipid extract (obtained from 2 g of sample) in a 250 mL flask. The flask was closed and heated for 30 min under nitrogen on a heating block (LTHS 250, Brnenska Druteva, Brno, Czech Republic). Then, freshly prepared 15% boron trifluoride in methanol (5 mL), was added to methylate the samples. After 2 min, heptane (5 mL) and sodium chloride (saturated solvent, 2 mL) were added and the sample was removed from the heating block. Next, heptane (15 mL) and sodium chloride (saturated solvent, 40 mL) were added to extract the FAMEs, the mixture was shaken and phases were separated and subsequently washed with sodium chloride (saturated solvent, 40 mL). The heptane phase was separated and anhydrous sodium sulfate was added. Quantitative determinations of FAMEs were conducted using a Shimadzu GC-2010 gas chromatograph (Shimadzu Corporation, Tokyo, Japan) equipped with a flame ionization detector (FID) and a HP-88 (Agilent Technologies, Englewood, CO, USA) capillary column (100 m × 0.25 mm, 88% cyanopropyl-arylpolysiloxane stationary phase with the thickness of 0.25 μm). The injection volume was 1.0 μL, the temperature of injection port was 250 °C with the split ratio of 1:100 and nitrogen was used as a carrier gas, temperature program was 80 °C/5 min, 200 °C/30 min, 250 °C/15 min. Identification of FAMEs was conducted by comparing their retention times with those of a 37 FAME reference standard. For quantification of FAMEs, methyl undecanoate was used as an internal standard. The FA results are expressed as a percentage of total FAMEs.

### 3.4. Statistics

The results of total lipids were expressed as means with standard deviations (SD) of each sample. Each sample was analyzed in triplicate (*n* = 3). Statistical differences among the samples were estimated by unpaired *t*-test and a probability value of *p* < 0.05 was considered to be statistically significant. Statistical analysis was performed using the StatPlus:mac LE Version 2009 software (AnalystSoft Inc., Atlanta, GA, USA). The analytical FA composition results are expressed as the average of six analyses (*n* = 6).

## 4. Conclusions

This study has examined nine commercially available edible cyanobacterial, microalgal and seaweed products and, moreover, autotrophically cultivated samples of the green microalga *Chlorella kessleri* and the cyanobacterium *Spirulina platensis*. Lipid content and FA profiles were determined using two different solvents, a mixture of methanol/chloroform/water (1:2:1, v/v/v, solvent I) and hexane (solvent II). In addition, yields of lipids and FA profiles after the extraction with different solvents were compared and, furthermore, comparison of data obtained from the determination of microalgal and cyanobacterial products and autotrophically cultivated microalga and cyanobacterium was accomplished.

Evidently, edible microalgal and cyanobacterial products contained a higher proportion of lipids than edible seaweed products using both solvent systems, and the highest lipid content was observed in autotrophically cultivated *C. kessleri* and *S. platensis*. From the lipid content point of view, the cultivated algae appear to be a better source of lipids than analyzed processed algal products.

The highest content of PUFAs, especially ω-3 FAs, was determined in the microalgal product of the green alga *C. pyrenoidosa* and two products of the brown seaweed *U. pinnatifida* (W, WI).

Even though fresh microalgae and unprocessed algae usually contain higher amounts of lipids, the dried edible microalgal product of *C. pyrenoidosa* examined in this work had a relatively high total lipid content and the highest level of PUFAs, especially ω-3 FAs. This investigation of edible cyanobacterial, microalgal and seaweed products and cultivated algae attested to the presence of health-promoting nutrients, such as PUFAs, especially essential ω-3 FAs, and this fact makes them a useful food supplement. 
